# First Autochthonous Report on Cattle *Babesia naoakii* in Central Java, Indonesia, and Identification of *Haemaphysalis bispinosa* Ticks in the Investigated Area

**DOI:** 10.3390/pathogens12010059

**Published:** 2022-12-29

**Authors:** Penny Humaidah Hamid, Muhammad Cahyadi, April Hari Wardhana, Dyah Haryuningtyas Sawitri, Nadya Nurvita R. Setya, Titis Insyariati, Heri Kurnianto, Carlos R. Hermosilla

**Affiliations:** 1 Department of Animal Science, Universitas Sebelas Maret, Surakarta 57126, Indonesia; 2 Research Center for Veterinary Science, National Research and Innovation Agency, Bogor 16114, Indonesia; 3 Institute of Parasitology, Justus Liebig University Giessen, 35390 Giessen, Germany

**Keywords:** *Babesia naoakii*, *Haemaphysalis bispinosa*, clinical babesiosis, tick-borne diseases

## Abstract

In tropical countries, clinical bovine babesiosis is a tick-borne disease primarily caused by *Babesia bovis* and *Babesia bigemina*. Here, we investigated 11 cattle with presumptive diagnosis of clinical babesiosis in Boyolali district, Central Java, Indonesia. The majority of the animals were anemic, as evidenced by lower hematocrit, hemoglobin concentration, and red blood cell counts than the normal ranges. Blood DNA was analyzed by a PCR assay targeting the 18S rRNA-ITS region of babesial origin, and the results confirmed that the cattle were infected with *Babesia* species. The sequencing and phylogenetic analyses demonstrated that the animals were infected with *Babesia naoakii*. This is the first report of *B. naoakii* in Indonesia and of *B. naoakii*-induced clinical bovine babesiosis outside of Sri Lanka. *B. naoakii* causes a persistent infection, as indicated by positive PCR results for serial blood samples of the circulatory system taken two weeks after treatment. Consequently, subclinical or newly recovered cattle may serve as potential intermediate hosts and infect ticks as definitive hosts to complete the life cycle. To identify potential tick vectors, we collected ticks from cattle, including 11 animals with clinical babesiosis. Based on the morphology and the mitochondrial cytochrome c oxidase subunit 1 (COX1) of collected ticks, we found that all of the collected ticks were *Haemaphysalis bispinosa*, identifying this tick species as a potential vector of *B. naoakii* in Indonesia. In this study, the evaluation of local farmers’ awareness and practices regarding tick-borne diseases is presented, as disease prevention is also reliant on the implementation of strategies for vector control. Since livestock activities in Java represent the country’s busiest animal trade, thereby the spread of disease to other regions is possible through anthropogenic factors. In conclusion, *B. naoakii* is a causative pathogen of clinical bovine babesiosis autochthonously occurred in this report and further research on *B. naoakii*-infection is required in other regions of the country. The prompt treatment of the disease seemed crucial for animal survival, which implies the necessity of early diagnosis and a sensitive detection method.

## 1. Introduction

Animal health management is one the most important factors affecting optimum production in livestock husbandry worldwide. The animal health status of livestock is not only important for ensuring the economic success of the industry but also for public health safety issues, as the end products are destined for human consumption and the transmission of zoonotic diseases should thus be avoided. The effectivity of livestock production is focused on how to produce in a profitable context and, nowadays, by considering an environmentally safety system for long-term, sustainable production. Cattle are one type of livestock that contribute to fulfilling the national dietary protein requirements in Indonesia; however, the total meat production declined by 6.81% in 2020 [1]. In order to ensure food security, food self-sufficiency, and national food resilience, the President of Indonesia established the National Food Agency (Bapanas) and issued Presidential Regulation No. 66 in 2021. This is due to the fact that 98% of cattle rearing in the community is still traditionally managed, resulting in poor daily cattle weight gain and the development of diseases [1]. Given the tropical and humid climate of Indonesia, the risk of infection with pathogenic agents is exacerbated. Indeed, animal diseases, including ecto- and endoparasites of the tropics, are among the major constraints in such efforts.

Babesiosis is a common tick-borne parasitic disease (TBD) occurring in wild and domestic mammals [2]. The disease is caused by various apicomplexan protozoan species of the family Babesiidae, order Piroplasmida, genus *Babesia*. Cattle babesiosis results in significant morbidity and mortality in domestic cattle. *Babesia bovis* and *Babesia bigemina* are the most prevalent species in tropical and subtropical climates, including Asia, Africa, Central and South America, parts of Southern Europe, and Australia [3]. Conversely, *Babesia divergens* and *Babesia major* are present in some temperate areas of Europe and North Africa [4,5]. Additionally, *Babesia naoakii*, formerly known as *Babesia* sp. Mymensingh, is clinically manifested in cattle of Sri Lanka. It has been detected subclinically in Vietnam, Bangladesh, and Mongolia among cattle and has been reported in camels of Egypt [6,7,8,9].

Ixodid tick vectors, which act as definitive hosts, transmit *Babesia* species to their intermediate vertebrate hosts [4]. Approximately 78% of all known tick species are members of Ixodidae, which is the most economically significant tick family worldwide. Importantly, ixodid ticks include the genera *Ixodes*, *Dermacentor*, *Rhipicephalus*, *Haemaphysalis*, *Hyalomma*, and *Amblyomma*. *Rhipicephalus* (*Boophilus*) *microplus*, a common tick species in the tropics and subtropics, is the primary vector for *B. bigemina* and *B. bovis*. Other significant ixodid tick genera transmitting cattle babesiosis also include *Haemaphysalis* and multiple *Rhipicephalus* species [10].

Both cattle babesiosis and ticks have significant adverse effects on the cattle industry, with high economic losses, as affected animals cause decreases in meat and milk production. In addition, substantial economic losses can be attributed to animal deaths and the high costs of tick management/control, diagnosis, prophylaxis, and treatment [4]. Tick infestation has both direct and indirect negative effects on animal production. Tick-derived anemia, tick toxicosis, severe allergic reactions to tick saliva, chronic stress, and skin irritability are direct negative effects resulting in immunosuppression that thereby affect animal health status. Examples of indirect losses are inefficiencies in the production chain of milk/meat, the costs of cattle babesiosis clinical treatment and acaricide treatment, the adverse ecological effects on the environment due to constant acaricide usage, acaricide resistance development, and trade restrictions on animal products among potential trading partners [11].

Several African and Asian countries, including Africa, Uganda, Pakistan, China, and Ethiopia, presently consider TBDs as the most significant animal disease threat. *B. bovis* and *B. bigemina* are *Babesia* species reported in cattle in Indonesia, with positivity rates of nested PCR were 50.7% for *B. bovis* and 19.1% for *B. bigemina* [12]. This surveillance analysis was conducted of random sampled species crossing the archipelago which is as the most comprehensive report to date [12]. To the best of our knowledge, no further investigations on the possible presence of other bovine *Babesia* species have yet been conducted. Currently, there are still very limited data on the prevalence, distribution, risk factors, and vectors with respect to time and region. In this report, we present, for the first time, an autochthonous case of *B. naoakii* infection in the central region of Java, Indonesia, with evidence of clinical manifestation and presence of tick, *Haemaphysalis bispinosa* in the investigated area.

## 2. Materials and Methods

### 2.1. Ethical Clearance

The experiments related to blood sample collection in this study were approved by the Ethical Committee of the Ahmad Dahlan University (No. 022206034), Yogyakarta, Indonesia. All the farmers participating in this study were informed about the investigation purposes and thereafter approved procedures of animal blood collection through the jugular vein. The farmers also voluntarily helped in restraining animals for blood collection as well as in tick observations and tick collections around their premises.

### 2.2. Study Location

**Figure 1:**
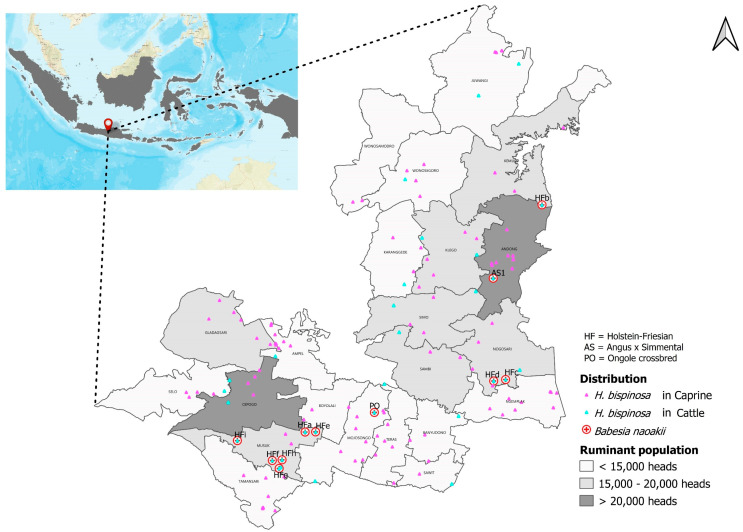
The sampling area is composed of 22 subdistricts in Boyolali, Central Java, Indonesia.

The Boyolali district is located in the middle of Central Java Province, Indonesia, with eastern longitudes of 110°36′9″ E and southern latitudes of 7°31′55.92″ S as geographic coordinates (Figure 1). Geographically, the Boyolali district is located in the center of Java Island. The data for distribution of blood parasites were transformed into a map using an open-source QGIS software, version 3.10.4-A Coruña, available online: https://qgis.org/en/site/, accessed on 2 October 2022).

### 2.3. Animals

Information on the cattle breed, age, gender, and animal health status was recorded before blood sample collection. The blood was collected from the jugular veins of cattle using a sterile 3 mL syringe. Animal handling procedures to avoid stress were implemented by the local farmers and Indonesian veterinarians. All the included cattle from which blood was collected had no reports of previous piroplasm vaccination, as a piroplasm vaccination program has never been conducted in Indonesia. The blood samples (*n* = 11) were collected from cattle showing marked hemoglobinuria during the investigation. Pathophysiological conditions such as fever and tachypnea were observed during sampling in this animal cohort (*n* = 11). Cattle with clinical signs were treated with anti-protozoal agents (please refer to Table 1) by local veterinarians at the time of blood collection. A second blood collection procedure was performed in 7 out of 11 cattle. Blood was stored in an EDTA vacutainer and transported with an ice pack to the Central Laboratory Unit, Universitas Sebelas Maret, Indonesia. Hematological analyses were performed using an automatic veterinary hematology analyzer (Mindray, Shenzhen, China) for hemoglobin, erythrocytes, and hematocrit values. All the remaining blood samples were stored at −20 °C and, thereafter, total DNA was extracted.

### 2.4. Total DNA Extraction of Cattle Blood Samples

Total DNA extraction was performed using the Genomic DNA mini kit for blood (Geneaid, Taiwan) according to the manufacturer’s instructions. A total volume of 300 µL of each blood sample was processed for DNA extraction under sterile laminar flow chamber conditions. The DNA was stored at −20 °C until further use.

### 2.5. PCR Amplification for Babesia sp.

PCR amplifications were performed to amplify the specific ITS1 DNA region of *Babesia* sp., subjected to sequencing [12,13]. ITS1 DNA was amplified in 25 µL total reaction buffer with the following composition: 1 µL total DNA, 1 µL forward primer 5′CGTCCCTGCCCTTTGTA3′, 1 µL reverse primer 5′TATTTTCTTTTCTGCCGCTT3′, 12.5 µL Bioline HS Master mix, 6.5 µL H_2_O. The PCR amplification was performed according to previously described protocols [12] and consisted of denaturation for 5 min at 94 °C, 35 cycles of a second denaturation step at 94 °C (1 min), an annealing step at 60 °C (1 min) for *Babesia* sp., and extension at 72 °C (1 min), and then a final extension at 72 °C for 10 min.

### 2.6. Nucleotide Sequencing of Babesia sp. ITS 1 DNA

PCR amplicons from eight DNA samples were sequenced by a commercial company (LPPT UGM, Indonesia and First Base, Singapore). All the sequences were then analyzed by trimming and assembly using Geneious Prime (Biomatters, New Zealand). Only 386 bp nucleotides were analyzed, with clear nucleotide peaks, and four isolates submitted to GenBank.

Phylogenetic tree was constructed from the *B. naoakii* ITS regions obtained in this study, along with corresponding sequences from other *Babesia* sp. obtained in genetic databases based on the Tamura–Nei model [14]. The analysis involved 17 nucleotide sequences. All positions containing gaps and missing data were eliminated. There were 253 positions in the final dataset. The genetic relatedness was analyzed by maximum likelihood phylogenetic tree using MEGA7 [15]. Bootstrap test with 1000 reiterations was used to estimate the confidence of the branching pattern of phylogenetic trees.

### 2.7. Tick Collection

Tick collection was performed manually, wearing gloves, with tick twisters during the sampling period. All collected ticks originated from naturally infested goats (*n* = 340) and cattle (*n* = 500) in the sample geographic area. Ticks were stored individually in absolute ethanol for further molecular, morphometric/morphological light microscopy, and scanning electron microscopy (SEM) analyses.

### 2.8. Tick Identification

The tick isolates were soaked in eugenol before being morphologically identified using a light microscope (Olympus, Japan) based on taxonomical keys. Additionally, ticks were stored in glutaraldehyde and sent for further processing analysis under SEM (JEOL JSM-6510LA, USA) at the PUI Baterai Universitas Sebelas Maret, Indonesia.

Molecular identification of ticks was based on PCR of the Ixodidae mitochondrial COX1 gene. Total DNA was individually extracted from ticks utilizing the sterile micropestle provided in the Genomic DNA mini kit^®^ for tissue (Geneaid, Taiwan), according to the manufacturer’s instructions. Primer pairs for the molecular identification of ticks were used based on the COX1 gene of the family Ixodidae [16]. The composition of PCR mixes was as follows: 1 µL total DNA, 1 µL forward primer 5′GGAACAATATATTTAATTTTTGG3′, 1 µL reverse primer 5′CTATCCCTACTGTAAATATATG3′, 12.5 µL Bioline HS Master mix, 6.5 µL H_2_O. In order to obtain specific-single PCR bands, a touchdown program was conducted as follows: denaturation at 94 °C for 15 min, followed by 10 cycles of denaturation at 92 °C for 1 min, annealing at 42 °C for 1 min, and elongation at 72 °C for 1 min. Afterwards, the program followed 32 cycles of denaturation (92 °C, 1 min), annealing (46 °C, 35 s), and final elongation (72 °C, 1 min). Nucleotide sequencing of Ixodidae COX1 genes was performed on 8 isolates (LPPT UGM, Indonesia). Sequences were analyzed and found to be 857 bp in length. Seven sequences were deposited in GenBank.

Phylogenetic trees were constructed from the COX1 of *H. bispinosa* in this study along with corresponding sequences banked in genetic databases based on the Tamura–Nei model [14]. The analysis involved 28 nucleotide sequences with 533 positions in the final dataset. The genetic relatedness was analyzed by maximum likelihood phylogenetic tree using MEGA7 [15] with 1000 Bootstrap reiterations

### 2.9. Farmer Questionnaire

In total, 96 farmers from the sampling area were interviewed during this study. Some of the farms were termed “group farms”, and their animals were located within a single subdistrict but owned by different farmers. Although located in the same “group farms”, responsibilities for cattle were attributed to each farmer for their exclusively owned animals. Therefore, the respondents were the main persons responsible for cattle-related activities in these farms, i.e., feeding, cleaning of litter, and other aspects of cattle management. All participating farmers also gave responses to the questionnaire concerning their knowledge of arthropod-borne diseases. Before any farm activities, the respondent was informed of the objectives of the current study; thereafter, farmers read and signed a document confirming their agreement to participate in the voluntary study. Profiles of farmers’ responses to the questionnaire are presented in Table 2.

## 3. Results

### 3.1. Clinical Manifestations of Cattle and Tick Identification

Clinical hemoglobinuria was found in all affected cattle (Table 1, video in Supplementary Materials). However, fever was not always detected in the cattle during the onset of hemoglobinuria. Hypoxia, which corresponds to an anemic condition, was indicated in six cattle with tachypnea (i.e., higher respiration rates) and posterior recumbency. The eleven animals showing hemoglobinuria from the beginning of this study received a single-dose injection of an anti-protozoal drug by local veterinary surgeons during the sampling period (Table 1). The single anti-protozoal agent therapy was markedly more expensive than antibiotics for the treatment of bacterial infections in the surveyed area. The local veterinarians mentioned that the prices of single injections and services against cattle babesiosis were five times higher than for antibiotics due to the high costs of drugs in the geographic area of the study. In this report, all clinically manifested cattle babesiosis cases received only single injections for therapy. The cattle babesiosis therapy can frequently fail due to the late phase of infection, i.e., high parasitemia, and advanced pathogenesis is indicated by severe anemia, panting, fever, collapse, and the presence of hemoglobin in urine (hemoglobinuria), changing from light red to blackish red on cattle HFb (Table 1). Some farmers were reported to have sold their *Babesia*-infected cattle immediately after the disappearance of hemoglobinuria. Therefore, only seven animals were subjected to a second blood collection after 2 weeks post-treatment. Clinical cases were observed several days post-treatment after single injection and the blood of seven animals was sampled. Five recovered animals were still PCR-positive after 14 days post-treatment.

The ticks collected from cattle surveyed in the Boyolali district were identified as *H. bispinosa* based on morphological characterization (see Figure in Supplementary Materials) according to a previous description. The distribution of ticks collected from cattle and goats is presented in Figure 1. For tick identification purposes, 200 out of 374 (55%) specimens collected from cattle and 200 out of 500 (40%) collected from goats were analyzed. The animals with hemoglobinuria and harboring *H. bispinosa* were also included, reaching an occurrence of 91% (10/11). It is important to mention that only *H. bispinosa* species were isolated from all cattle surveyed in the Boyolali district of Java, Indonesia.

### 3.2. PCR Assays for Babesia and Ixodidae

**Figure 2:**
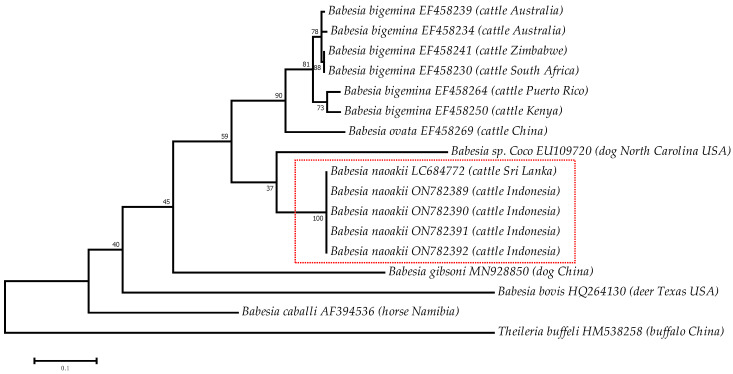
Phylogenetic analysis of *B. naoakii* from Central Java based on ITS1 region. Maximum likelihood phylogenetic tree based on internal transcribed spacer 1 (ITS1). Phylogenetic tree was reconstructed with the Tamura–Nei model with 1000 bootstrap reiterations. The isolates from this study are given within the box.

BLAST nucleotide analysis revealed the sequences shared 99.69% identity with the corresponding sequences of isolate previously reported in Sri Lanka, i.e., LC684772.1. Moreover, the sequence length for phylogenetic construction was 321 bp due to the lack of the same fragment of small subunit ribosomal RNA-ITS1 with reference sequences stored at NCBI. Four isolates from this study were positioned in the same clade as *B. naoakii* previously reported by Sivakumar et al. (2022) (Figure 2).

**Figure 3:**
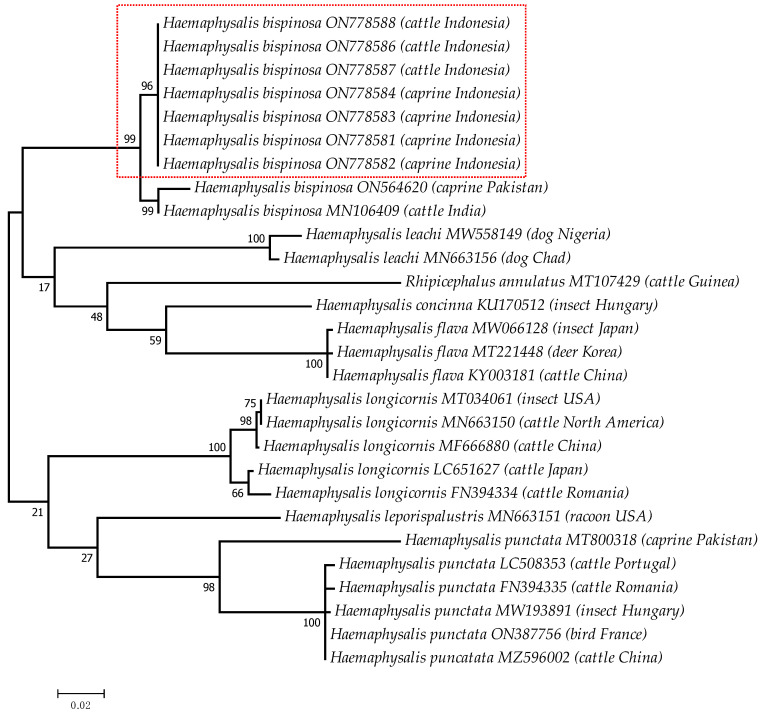
Maximum likelihood tree for *H. bispinosa* from Central Java based on COX1. Phylogenetic tree was reconstructed by Tamura–Nei model with 1000 bootstrap reiterations. The isolates from this study are given within the box.

In total, 53% (200/374) of cattle and 40% (200/500) of caprine in this survey were infested with *H. bispinosa* ticks. Moreover, *H. bispinosa* ticks were collected from almost all of the animals suffering from hemoglobinuria, i.e., 91% (10/11). The COX1 fragments were 847 bp in length, and their sequences were submitted to NCBI with the accession numbers ON778581-ON778584, ON778586-ON778588, respectively, and shared 97–99% identity with another *H. bispinosa* species, mainly recorded in Asia. *H. bispinosa* sequences in this report were closely related to the same species isolated from caprine in Pakistan and cattle of India (Figure 3).

### 3.3. Knowledge of Arthropod-Borne Diseases among Farmers in the Boyolali District

Almost all farmers in this study, i.e., 95% (91/96), had some knowledge that their cattle might become infested by blood-sucking ectoparasites, and that these invertebrates could transmit diseases (15.63% (15/96)) to cattle (Table 2). The same was true regarding their awareness of parasite-derived hemoglobinuria during cattle husbandry. In total, 15.63% of the farmers (15/96) engaged in frequent cattle selling within 3 months. This is related to the fact that local cattle farmers in the Boyolali district generally purchase young calves, fatten them thereafter within several months, and finally sell them at higher prices. Farmers engaged in fattening activities mentioned that almost all cattle of market origin had no indication of ectoparasite infestations. Purchased cattle had already been bathed with acaricides to minimize apparent ectoparasite infestations when sold in local markets, therefore fetching higher prices than cattle with manifestation of ectoparasite infestation. The farmers mentioned that they had certain knowledge of common ectoparasites such as ticks and lice; however, they did not know how to differentiate them. Interestingly, flies were considered as the major ectoparasite concern by cattle farmers over tick, mite, and/or lice infestations. Local farmers frequently bathed their cattle and manually picked visible ectoparasites (mainly ticks) from the skin. Concerning anti-ectoparasite treatments, 94% of participating farmers (90/96) mentioned using acaricides/insecticides in spray form or consulting local veterinarians for acaricide/insecticide treatments via injection. However, local farmers had no knowledge of the active substances used for these treatments. Based on their experience, 92% (89/96) of respondents mentioned that there was usually recrudescence of ectoparasite infestations after treatments. The farmers believed that ectoparasite infestations were mainly occurring within their environment or premises during cattle husbandry, i.e., caused by flies, mosquitos, midges, blackflies, tabanids, and ticks flying around in or inhabiting the area, and that ectoparasites were not transmitted during cattle transportation from local markets. Nonetheless, none of the questioned farmers had found free-living ticks outside of the cattle body and/or had knowledge of either exogenous life cycle stages (e.g., larvae, nymphs) or the prolonged environmental fasting periods of ticks.

## 4. Discussion

In tropical and subtropical regions, the highly pathogenic species *B. bovis* and *B. bigemina* are known to cause high economic losses in cattle husbandry due to cattle babesiosis. Indonesia is known to be endemic for several TBDs, i.e., hemoparasites (*Theileria*, *Babesia*) as well as *Rickettsia, Ehrlichia,* and *Anaplasma* species in small and large ruminants. An update on the current status of these TBDs is limited due to the difficulty of animal sampling within the Indonesian Archipelago, composed of more than 17,000 islands with different flora, fauna, demography, and ecological biomes. To the best of our knowledge, this study represents the first report on the occurrence of an autochthonous cattle *B. naoakii* infection in the central region of Java Island in Indonesia. Moreover, the presence of *H. bispinosa* ticks in the investigated area was reported, as well as the evaluation of local farmers’ awareness of TBD, which is an important aspect considering the prevention of disease spread to non-endemic regions.

In this report, 10 out of 11 cattle showed clinical signs of babesiosis, such as severe hemoglobinuria, in Holstein-Friesian cattle (Table 1). The susceptibility of the HF breed to *B. naoakii* infection is currently unknown; the same holds true for the pathogenicity and immunity, and further detailed investigation into both is required. Cattle susceptibility to *Babesia* sp. with clinical manifestations has been reported in a 2-day-old, 6-day-old, less than 14-day-old, and suckling calves that were 15 days old [17,18,19,20]. *Babesia* sp. infections to these calves are through intrauterine transmission or by non-mothers after birth when the immune system is not developed enough [5]. *Babesia* infection in young cattle is supported by their softer and thinner skin, which facilitates a successful blood meal even for immature stadia of ticks [21,22,23]. The pathogenicity of *B. naoakii* in parasitized cattle was proven in this report as animals manifested severe hemoglobinuria, tachypnea, and fever. Almost all *Babesia*-infected cattle showed severely affected values for hemoglobin, hematocrit, and erythrocyte counts when comparing with reference values (Table 1). These adverse effects are most likely due to intraerythrocytic multiplication of all the genus of *Babesia*. However, detailed information about *B. naoakii* virulence and pathogenesis are still unknown. Since these cases were incidental, there were insufficient anamneses for these cases. However, all *Babesia*-infected animals were born within the investigated area and had not been imported from abroad, thereby confirming the autochthonous nature of the infection. The death of the *B. naoakii*-infected animals (Table 1) occurred when the farmer consulted local veterinarian authorities for assistance at the latest phase of infection, i.e., when the urine was already blackish red, the animal showed panting respiration and was unable to stand. It is noteworthy that the *B. naoakii*-infected animals died despite receiving proper anti-protozoal treatment, indicating that the early diagnosis of the disease seems crucial for achieving therapeutic success. Thus, therapies must be administered as soon as possible after the diagnosis and onset of babesiosis; otherwise, the animal cannot survive, as parasitemia and lethal outcomes will increase in likelihood [24,25]. Local treatments for cattle babesiosis in this study area included the usage of diminazene aceturate and phenazone, included in Tryponyl^®^ and Imidox^®^, which contain imidocarb dipropionate as effective anti-protozoal agents. Similarly to tropical *B. bovis* and *B. bigemina* species, *B. naoakii* seems to be highly pathogenic, showing detrimental effects on the cattle husbandry status in general, not only due to lethal outcomes and animal loss but also due to the maintenance health costs of anti-protozoal therapy.

Tick infestation was not always observed in *Babesia*-infected animals during blood collection. In total, 200 out of 374 ticks collected from cattle and 200 out of 500 caprine ticks corresponded to *H. bispinosa*. The distribution of *H. bispinosa* in the Boyolali district also confirms previous reports of its sole distribution in the Special Region of Yogyakarta and Riau Provinces, whilst eastern parts of Indonesia are mainly populated by *R. microplus* [26]. *H. bispinosa* is well known to act as a potential vector of various *Babesia* sp. [23]. Moreover, *H. bipinosa* is distributed in South Asian countries, such as Pakistan [27], India [28,29], Bhutan [30], Bangladesh [31], Peninsular Malaysia [32,33,34], Thailand [35], Singapore [36], Laos [37], and Sri Lanka [38], and was subsequently introduced to Borneo [39]. The species is closely related to *H. longicornis*, which is distributed in more temperate regions such as Australia, New Zealand, New Caledonia, the New Hebrides, Fiji, Japan, Russia, Korea, and China and has now been introduced to vast areas of the world [40,41]. In this report, the sequence of the COX1 gene of *H. bispinosa* was found to be nearly identical to those of ticks collected in Pakistan and India (Figure 3), which may imply that *H. bispinosa* has spread through the route of South Asia, from Peninsular Malaysia to the western part of Indonesia, i.e., Sumatra and Java. Concerning the transmission of cattle babesiosis in Indonesia, it is assumed that *R. microplus* ticks are still the only vector playing a pivotal role in the transmission of cattle babesiosis [12]. In this report, detection of *Babesia* sp. from *H. bispinosa* utilizing primer pairs for blood samples showed that 100 pooled ticks collected from 10 infected cattle, fed and unfed, were positive for *Babesia*. In this report, we hypothesize that the presence of *H. bispinosa* represents the highest risk factor for the transmission of *B. naoakii* to this animal. Moreover, *H. bispinosa* might act as a potential vector of other relevant ruminant pathogens, but further investigations on this aspect are needed. The vector competency of *H. bispinosa* ticks to propagate *B. naoakii* needs to be investigated, including experimental animal studies. Despite *H. bispinosa* having parthenogenic reproduction capacities that may influence its spread capability into previous non-endemic regions, growing concerns are linked to investigations performed in neighboring countries on its zoonotic microbiome properties [33,34,36]. Given that the area is densely populated, this may offer possibilities for investigations on the spillover of the tick microbiome into human populations in the Boyolali district. Consequently, TBDs are still considered as emerging as well as neglected diseases worldwide and thus require constant investigation efforts focusing on their complex epizootiology and considerations related to being within the One Health framework.

The knowledge on tick biology, epidemiology, pathogenicity, immunity, and their critical role as potential vectors of emerging or re-emerging zoonotic pathogens, including bacteria, virus, fungi, and parasites, has increased, but further investigations in the tropics, in areas with high biodiversity, are needed. Due to the growing concern that *B. naoakii* can cause severe clinical babesiosis in cattle, this species is currently experiencing a high level of attention and interest within the parasitology community. As shown here, *B. naoakii* causes persistent infection, as indicated by positive PCR results in serial blood samples taken two weeks after treatment, indicating their persistence in the circulatory system. Consequently, subclinical cattle carrying *B. naoakii*-infected erythrocytes may serve as potential intermediate hosts (reservoirs) and infect new ticks that then act as definitive hosts, wherein the occurrence of gamogony and sporogony results in completion of the life cycle. Overall, additional research on *B. naoakii*-induced babesiosis is required both in Indonesia and elsewhere in tropical Asia to identify new endemic regions. Since the region is one of the most populous in terms of domesticated cattle, small ruminants, and the presence of ticks, the spread of this novel species is highly likely. The island of Java still represents the busiest area of the country’s animal trade, thereby possibly contributing to the spread of disease through anthropogenic factors. Anthropogenic factors are known to significantly impact the epidemiology of blood-sucking ectoparasites, including ticks. Consistently, hematophagous ectoparasites were also detected in imported feeder cattle [42] and have been, for a long time, present in Indonesian territories [43]. All piroplasm parasite genera, i.e., *Theileria* and *Babesia*, must be investigated from the definitive host perspective (i.e., ticks), as their control seems to be essential for preventing the spread of babesiosis. Other livestock, such as sheep and goats, which can also serve as natural reservoirs for diseases with health and economic significance, have not yet been investigated in the Boyolali district and will be addressed in the near future.

The results of this study also demonstrate that local farmers still lack adequate knowledge on TBDs, resulting in inadequate attitudes toward proper vector control. This implies that efforts must be taken regarding the education of not only local farmers but also of public health authorities on TBDs, ticks, risk factors, vector control, prophylaxis, and prevention schemes.

## 5. Conclusions

*Babesia naoakii* infection manifested as clinical babesiosis in central region of Indonesia. *B. naoakii* caused a persistent infection, as indicated by positive PCRs by serial blood samples taken two weeks after treatment. This study also reported the presence of ticks, *H. bispinosa*, in the investigated geographic area. The TBDs are considered as neglected diseases worldwide and thus demanding constant investigation effort not only due their vectorial capacity to animal diseases but also human in the considerations within One Health. This study also showed that the farmers still lack adequate knowledge on TBDs. This condition implies that efforts must be taken in an integrative approach on the prophylaxis, treatments, vector control strategies, and a proper transfer knowledge to locals.

## Figures and Tables

**Table 1:** Clinical signs, hematological profiles, medication administered by local veterinary services, and PCR results of cattle clinically affected by *B. naoakii* in Indonesia.

No.	Breed	Age	hb	rbc	hct	Treatment	1st Observation		2nd Observation	
		(Years)	(g/dL)	(×10^6^/uL)	(%)	(Single Injection)	1st PCR	Hemoglobinuria	2nd PCR	Hemoglobinuria
1	AS	0–2	9.6	7.9	22.1	imidocarb dipropionate	positive	observed	positive	not observed
2	HFa	0–2	6.3	6.2	19.2	imidocarb dipropionate	positive	observed	positive	not observed
3	HFb	0–2	5.6	4.5	16.5	imidocarb dipropionate	positive	observed	negative	not observed
4	HFc	0–2	6.1	5.8	15.1	diminazene aceturate, phenazone	positive	observed	positive	not observed
5	HFd	0–2	8.9	7.4	21.2	diminazene aceturate, phenazone	positive	observed	sold	not observed
6	HFe	0–2	3.4	2.1	5.3	diminazene aceturate, phenazone	positive	observed	dead	not observed
7	PO	2–6	4.9	4.6	9.7	imidocarb dipropionate	positive	observed	positive	not observed
8	HFf	2–6	6.7	7.5	15.1	imidocarb dipropionate	positive	observed	positive	not observed
9	HFg	2–6	7.1	6.1	18.9	diminazene aceturate, phenazone	positive	observed	sold	not observed
10	HFh	2–6	6.9	5.5	12.1	imidocarb dipropionate	positive	observed	sold	not observed
11	HFi	2–6	6.4	6.2	13.2	imidocarb dipropionate	positive	observed	negative	not observed

hb: hemoglobin; rbc: red blood cell; hct: hematocrit; normal blood values: hb 8–15 g/dL, rbc 5–10 × 10^6^/µL, hct 24–46% (Merck Veterinary Manual). AS: Angus Simmental cross; HF: Holstein-Friesian; PO: Peranakan Ongole (Ongole cross unspecified breed). ID a, b, c, d, e, f, g, h, i correspond to animal location in the investigated area (Figure 1). Local veterinarians treated these infected cattle with imidocarb dipropionate (Imidox^®^) or diminazene aceturate and phenazone (Tryponyl^®^) injection. The farmers usually sell infected cattle as soon as no hemoglobinuria signs are observed to minimize the risk of reinfection.

**Table 2:** Farmers/owners’ knowledge and activity related to arthropod-borne diseases in Boyolali district.

Parameters		Knowledge about Arthropod-Borne Diseases (*n*)
Cattle holding per household	<5	25
	5–10	61
	>10	10
Gender	Male	78
	Female	18
Age of the farmers	<20	2
	20–40	73
	>40	21
Frequent activity in selling cattle	1–3 month	15
	3–12 month	55
	>12 month	26
Knowledge of hematuria during cattle husbandry	Yes	15
	No	81
Understanding of arthropod-borne diseases	Yes	91
	No	5
Use of acaricide/insecticide substances	Yes	90
	No	6

## Data Availability

Data is contained within the article or supplementary material.

## Supplementary Materials

The following supporting information can be downloaded at: https://www.mdpi.com/article/10.3390/pathogens12010059/s1, Figure S1: Identification of tick morphology. Video S1: Clinically observed hemoglobinuria in Holstein-Friesian cattle.
