# Supplementary material for: First Autochthonous Report on Cattle Babesia naoakii in Central Java, Indonesia, and Identification of Haemaphysalis bispinosa Ticks in the Investigated Area
Source: Pathogens. 2022 Dec 29;12(1):59. doi: 10.3390/pathogens12010059 (PMC9864747; doi:10.3390/pathogens12010059)
Supplement: Supplementary file 1 [file pathogens-12-00059-s001.zip › Hamid_Suppl material_Fig_1.pdf]

Supplementary materials

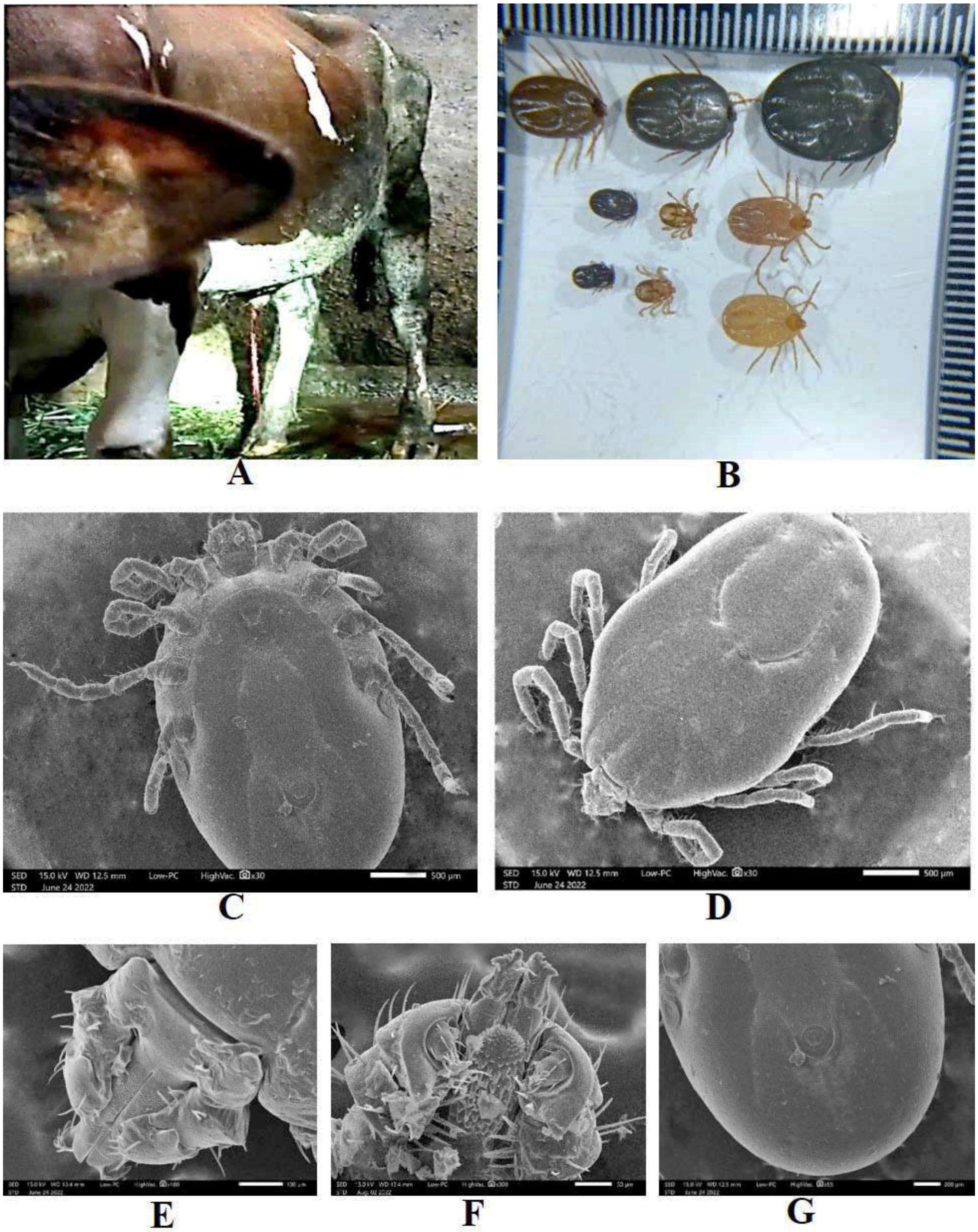

Figure 1. Clinical signs associated with bovine babesiosis in Boyolali, Central Java, and ticks randomly found during blood collection. A Holstein-Friesian calf with hematuria (hemoglobinuria) (A). *H. bispinosa* in various stages (B). Scanning electron microscopy of a tick, dorsal view (C), ventral view (D), gnathostome (E) and hypostome (F), ventral posterior view (G)
